# Gaussian Blurring Technique for Detecting and Classifying Acute Lymphoblastic Leukemia Cancer Cells from Microscopic Biopsy Images

**DOI:** 10.3390/life13020348

**Published:** 2023-01-28

**Authors:** Tulasi Gayatri Devi, Nagamma Patil, Sharada Rai, Cheryl Sarah Philipose

**Affiliations:** 1 Department of Information Technology, National Institute of Technology Karnataka, Mangalore 575025, India; 2 Department of Pathology, Kasturba Medical College, Mangalore 575001, India

**Keywords:** image processing, Acute Lymphoblastic Leukemia, Gaussian blur, Hue Saturation Value, cancer cell, ALL-IDB1

## Abstract

Visual inspection of peripheral blood samples is a critical step in the leukemia diagnostic process. Automated solutions based on artificial vision approaches can accelerate this procedure, while also improving accuracy and uniformity of response in telemedicine applications. In this study, we propose a novel GBHSV-Leuk method to segment and classify Acute Lymphoblastic Leukemia (ALL) cancer cells. GBHSV-Leuk is a two staged process. The first stage involves pre-processing, which uses the Gaussian Blurring (GB) technique to blur the noise and reflections in the image. The second stage involves segmentation using the Hue Saturation Value (HSV) technique and morphological operations to differentiate between the foreground and background colors, which improve the accuracy of prediction. The proposed method attains 96.30% accuracy when applied on the private dataset, and 95.41% accuracy when applied on the ALL-IDB1 public dataset. This work would facilitate early detection of ALL cancer.

## 1. Introduction

Acute Lymphoblastic Leukemia (ALL) is the most common cancer around the world. ALL originates in the bone marrow and is known to be a blood cell cancer, it is an uncontrolled rapid growth of immature blood cells that is seen mostly in children as well as in adults. After cardiovascular diseases, cancer is the leading cause of deaths (non-accidental) in the world, with leukemia being a common cause among blood diseases. Cancer is one of the world’s largest health problems, as the global burden of disease in 2017 estimates that 9.6 million people died prematurely as a result of cancer [1,2]. The global cancer survey of 2020 noted that leukemia caused 474,519 new cases and 311,594 deaths in that year [3]. Histopathological tests play an important role in the field of medicine and help diagnose the underlying disease [4,5]. Computer Aided Diagnosis (CAD) systems have been proposed for the segmentation, detection, and classification of various types of cancers, based on X-ray images [6,7].

There are well-established cancer detection approaches through different imaging techniques such as X-ray images, Ultrasound images, Positron Emission Tomography (PET), Magnetic Resonance Imaging (MRI), and Computed Tomography (CT), as well as pathological tests, namely, blood and urine tests, to assess and diagnose various types of cancers [8]. In contrast to the histopathological outcome of microscopic analysis of biopsy images, these are categorized into cancerous or non-cancerous tissues, based on the shape and size of the cell and nuclei, as well as the overall cell distribution.

There are two main types of leukemia: acute leukemia and chronic leukemia. Acute leukemia causes a rapid deterioration of the patient’s condition [9]. Chronic leukemia is a slow progressing condition that slowly, over a period of time, degrades the condition of the patient. Leukemia can be further classified into four types, based on the acute and chronic conditions, as Acute Lymphoblastic Leukemia (ALL), Acute Myelogenous Leukemia (AML), Chronic Lymphocytic Leukemia (CLL), and Chronic Myelogenous Leukemia (CML). Table 1 shows the survival rate of Leukemia-affected patients when the detection is made in the early stages. Diagnosing cancer at an early stage can improve survival rate. This motivated the proposed study.

Summarizing Contribution:

1. A comprehensive study has been conducted to explore the existing work. Most of the works concentrated on public datasets.
2. The proposed approach addresses the impact of pre-processing using Gaussian Blur Techniques, including reduction of noise and background removal, highlighting features that aid accurate classification.
3. HSV model with thresholding is used to segment the cancerous cells and draw contours around the segmented cells.
4. Used both public and private datasets to test the performance and compare with existing methods, which proves that the proposed method performs better.

The rest of the paper is structured as follows. Section 2 discusses the related works, the proposed method, and implementation details along with the dataset description chosen for this study. Section 3 discusses the results of the proposed work and their comparison with existing works. Section 4 draws the conclusions.

## 2. Materials and Methods

### 2.1. Related Work

Cancer is one of the leading causes of death throughout the world. Among all the cancer types, blood cancer, or leukemia, constitutes a significant proportion. Leukemia can be of acute and chronic sub types with acute lymphoblastic leukemia being the most common childhood blood cancer type [6,10]. This section of the paper analyzes different image processing techniques that have been used to detect cancer cells. In [11], a color thresholding algorithm-based Acute Lymphoblastic Leukemia (ALL) detection system is proposed, that firstly detects the white blood cells, segments the lymphocytes among the detected white blood cells, and then finally identifies and counts the lymphoblasts. An accuracy of 92.15%, sensitivity of 96.92%, and precision of 91.35%, were found for a sample size of 260 patient images. The proposed study compared two filters—Weiner filter and Median filter—and found the Median filter to be 8% better than the Weiner filter, when applied on private datasets.

In [12], the authors proposed a classifier based on Support Vector Machine (SVM) and Gradient Boosting Decision Tree (GBDT) for cancer cell detection based on geometric, texture, and statistical features. The dataset considered for the experiment was taken from The Cancer Imaging Archive (TCIA), the F1 score reported was approximately 86%. They are original images with illumination errors, and overlapping cell nuclei were fixed before conducting the experiment with GBDT and SVM. The specificity of the model was low, approximately 55%, which is a drawback.

Further to this, Khandekar et al., in [13], addressed the method based on You Only Look Once (YOLO) for classification and detection of leukemia cells from microscopic images with the ALL-IDB and C-NMC-2019 datasets. They were able to attain the results on the ALL-IDB dataset in terms of overall Mean Average Precision, Recall, and F1-score by 95.57%, 92%, and 92%, respectively, and on the C-NMC-2019 dataset with Mean Average Precision, Recall, and F1-score of 98.57%, 96%, and 92%, respectively. Furthermore, they proposed the YOLO-based methods to detect the blast cells.

Rastogi et al. [14] considered datasets, namely ALL-IDB, PBS-HCB, and AML, that were used to identify leukocytes by the proposed LeuFeatx model, derived from VGG16 and as a base model to classify with an accuracy of 96.5%.

The authors in [15] designed a new model based on an intelligent IoMT framework for classifying acute leukemia using microscopic images. The model includes three main steps: collect samples, push to a cloud server, and identify as either leukemia or healthy in cloud server. Finally, the obtained results are sent to a hematologist for medical approval and can act like a decision-making system. The best accuracy scores noted were 98.67% for binary classification and 95.5% for multi-class classification.

The authors in [16] proposed a simple, yet effective classification approach using a ResNeXt convolutional neural network, with Squeeze-and-Excitation modules. The approach was evaluated in the C-NMC online challenge and has achieved a weighted F1-score of 88.91% on the test set.

The authors in [17] proposed a deep convolutional neuro-fuzzy network, based on the Takagi–SugenoKang (TSK) fuzzy model for acute lymphoblastic leukemia diagnosis. The model achieved an accuracy of 97.31% on average.

The authors in [18] carried out a comparative analysis of different transfer learning models such as Xception, Inceptionv3, DenseNet201, ResNet50, and MobileNet, to detect Acute Lymphocytic Leukemia (ALL) from blood smear cells. All the models were trained on the ALL-IDB2 dataset and achieved an accuracy of 87.97%, 88.92%, 88.92%, 95.28%, and 97.88%, respectively.

The authors in [19] proposed two automated classification models based on blood microscopic images, to detect leukemia by employing transfer learning. They experimented without and with fine-tuning AlexNet—a pre-trained deep convolutional neural network model. Experiments were conducted on a dataset, consisting of 2820 images, confirming that the second model performs better than the first with 100% classification accuracy.

The authors in [20] used four different supervised ML algorithms, including Classification And Regression Trees (CART), Random Forest (RM), Gradient boosted Machine (GM), and C5.0 decision tree algorithm to retrieve the most useful discriminating attributes for pediatric acute lymphoblastic leukemia. Where, CART has proved to be the best and is a complete fit for the entire dataset yielding—it has a 99.83% model fit accuracy.

The authors in [21] presented an effective scheme for classification of the normal white blood cells from the affected cells in a microscopic image. The proposed data classification uses Discrete Orthonormal S-Transform (DOST) to extract the texture features, as its dimensionality is reduced, using linear discriminant analysis. The reduced features are then supplied to the proposed Adaboost algorithm with Random Forest (ADBRF) classifier, where the random forest is used as the base classifier. A publicly available dataset, ALL-IDB1, is used to validate the proposed scheme. The proposed method yields a superior accuracy of 99.66%, with five runs of k-fold stratified cross-validation, as compared to existing schemes.

The authors in [22] proposed a novel probability-based weight factor, which has a significant role in efficiently hybridizing MobilenetV2 and ResNet18, while preserving the benefits of both approaches. The proposed approach yielded the best accuracy of 99.39% and 97.18% in ALLIDB1 and ALLIDB2 public benchmark datasets, respectively. Results proved to be more efficient compared with recent transfer learning-based techniques.

The authors in [23] explored the weighted ensemble of deep CNNs to recommend a better ALL cell classifier. The weights are estimated from ensemble candidates’ corresponding metrics, such as F1-score, Area Under the Curve (AUC), and kappa values. Various data augmentations and pre-processing are incorporated to achieve a better generalization of the network. The proposed model is trained and evaluated utilizing the C-NMC-2019 ALL dataset and has resulted in a weighted F1-score of 89.7%, a balanced accuracy of 88.3%, and an AUC of 0.948 in the preliminary test set.

The authors in [24] worked on identifying ALL subtypes. They enhanced the images to reduce the brightness when converting from RGB to HSV, and then applied fuzzy c-means to segment the cores and separate them from the rest of the image. Further, the Random Forest (RF) classifier has been applied to classify L1, L2, L3, normal, reactive, and atypical cells. An accuracy of 98% was achieved when compared to two other common classifiers: MultiLayer Perceptron (MLP), and multi-SVM classifier, with more success, especially for recognition of L1, normal, and reactive cells.

The literature review provided information on cancer cell detection using image processing techniques. Certain statistics are discussed that provide the impact of medical image processing in the case of cancer detection and its effect in early detection. The various approaches used in medical image segmentation have been discussed. These techniques are effective in their fields and contribute significantly toward cell segmentation and the detection of cancer cells in medical applications. The importance of various pre-processing steps and the requirement of de-noising, for computer vision, is presented as part of this literature review. The research gaps are related to the accuracy of detection, the complexity of implementation, detection of many features or abnormalities, noise removal mechanisms, and limited use of real-time datasets.

### 2.2. Proposed Method

The study proposes a novel GBHSV-Leuk algorithm, based on Gaussian Blurring technique, Hue Saturation Value (HSV) technique and thresholding scheme, to accurately detect and classify Acute Lymphoblastic Leukemia type cancer cells from a microscopic biopsy image accurately.

- Gaussian Blur technique: This function is used to reduce the noise in an image. This can be done by applying the Gaussian function to blur the image. By blurring the image, the low spatial frequency of the image is preserved and the noise in the image is reduced by removing some of the unwanted details in the image. Equation (1) gives the Gaussian blur formula, where *X* is whichever direction the run is being made at the time [25] and σ is the standard deviation of the Gaussian distribution. This two-pass method of Gaussian filtering requires fewer calculations and, therefore, is generally preferable in implementation.
  (1) $$g\left(X\right)=\frac{e^{-\frac{x^{2}}{2\sigma^{2}}}}{\sqrt{2\pi \sigma^{2}}}$$
- RGB Color Space: RGB model translates to Red–Green–Blue. The color images are formed by a combination of three images—red image, green image, and blue image. A color image matrix is formed by a combination of the red matrix, blue matrix, and green matrix, while a grayscale image is made up of only one matrix.
- HSV Color Space: The HSV (Hue, Saturation, and Value) color space is a representation of colors that is similar to how the human eye perceives it. Hue is a channel that encodes the color information when translated from RGB (Red–Green–Blue) and corresponds to 0 degrees for red, 120 degrees for green, and 240 degrees for blue. Saturation is a channel that encodes the color purity and intensity. Value is a channel that encodes color brightness, gloss, and shading.
- Morphological Operation Transformation: Morphological operations are applied to provide a structuring element to the image based on the comparison between the input image’s corresponding pixel with its neighbors. The morphological operations are dilation, erosion, opening operation, and closing operation [26].
- Binary Threshold: Thresholding is an image processing method to create a binary image by setting the thresholding value on the intensity of the pixel of the image.

### 2.3. Proposed Algorithm

The Algorithm 1 gives the step-by-step operations carried out. The image is loaded from the dataset. The original image is resized to fit the display window and decrease the memory usage. The loaded image has noise in it and has not been subjected to any image pre-processing techniques. The input for the algorithm is the image under the test for detection. The Gaussian blur technique is applied, and the formula is shown in Equation (1). The Hue Value (H) is computed per pixel, by using the largest values of the R, G, B values and are calculated as the highest value of the color using Equation (2). The HSV Color Model considering *max* to be the maximum of the (R, G, B) values, and *min* the minimum of those values.

(2) $$H=\left\{\begin{array}{c} \frac{\left(G-B\right)}{\left(max-min\right)},if\;m_{max}=R\\ 2.0+\frac{\left(B-R\right)}{\left(max-min\right)},if\;m_{max}=G\\ 4.0+\frac{\left(R-G\right)}{\left(max-min\right)},if\;m_{max}=B\\ H^{{}^{\prime }}=H*{Scale}_{h}\\ Undefined,\;if(max-min)=0 \end{array}\right.$$

**Algorithm 1** GBHSV-Leuk.**Input:** Microscopic Image

   **for** F(I${}_{t}$[m][n]) = I${}_{t}$[m][n] − I${}_{ut}$[m][n] **do**

      Apply Gaussian Blur technique

      **if**
F(n) is not filled **then**

         Fill pixel

      **end if**

      Convert the blurred frame from RGB color space to HSV color space

      Convert the hue value using the largest RGB values

      Compute the L${}_{b}$ and U${}_{b}$ hues

      Calculate I${}_{m}$, which is the mask image

      Create 5 × 5 kernel to remove noise

      Compute the structuring element *S*

      Compute the dilation function

      Apply binary threshold

      Compute detection using threshold value and transformation function

      Apply Bitwise AND between the Original image and Threshold

   **end for**

   Highlight the cancerous cell with contours

   **return**
**Output:** Image with cancerous cell highlight using contours

   **Notations in the algorithm:**

   I${}_{t}$ = Image under test

   m = Number of rows of image pixels

   n = Number of columns of image pixels

   L${}_{b}$ = Lower hue

   U${}_{b}$ = Upper hue/ higher hue

   I${}_{m}$ = Mask image
   *S* = Structuring element


Morphological transformation is applied on the image. The upper and lower bound of the Hue Saturation Value (HSV) is applied, followed by Image mask, where M_r_ is the mask in the range of L_b_ and U_b_. The formula for the calculation of lower bound is shown in Equation (3), the formula for the calculation of upper bound is shown in Equation (4), and the formula for the image mask is shown in Equation (5).

(3) $$L{}_{b}=L\left(I{}_{t}\left(m,n\right)\right)$$

(4) $$U{}_{b}=U\left(I{}_{t}\left(m,n\right)\right)$$

(5) $$I{}_{m,n}=M{}_{r}\left(L{}_{b},U{}_{b}\right))$$

There are certain unfilled pixels in the image, which are usually considered as features by the pre-processing techniques. It is important to fill and cover the unfilled pixels in the image so that the processing and segmentation techniques can be efficient. The Gaussian Blur method is applied so that the noise in the image is blurred in this step. Among the several blood parts of the image, the frames are converted from Red–Green–Blue (RGB) to HSV. Conversion is required because the RGB values only represent the luminance (intensity) that does not have color information. HSV has color information used to detect abnormalities. A one-time operation is used in which the details of an upper hue and lower hue are obtained. A mask is created in the next step, which distinguishes between the upper and lower HSV.

A kernel of size 5 × 5 is created. It is used as a filter to remove the noise. Morphological operations and transformations from the application of filters are used in this step to remove the noise from the image. The proposed method applies the Gaussian blur technique to filter the noise. This, however, also makes the edges of the image rugged due to the process. It is not suitable to have rugged edges in the image. Therefore, the process of dilation is used. The image isolation is given as I${}_{S}$ and the dilation of the image is given as D${}_{I}$. The erosion of a binary image f by a structuring element S (denoted f ⊖ S) produces a new binary image g = f ⊖ S, with ones in all locations (x,y) of a structuring element’s origin at which that structuring element s fits the input image f, i.e., g(x,y) = 1 is S fits f and 0 otherwise, repeated for all pixel coordinates (x,y). Equation (6) shows the formula for the calculation of the structuring element and the formula for the dilation of the image is shown in Equation (7).

(6) $$g\left(I{}_{t}\left(m,n\right)\right)=f\left(I{}_{m},k\left(5,5\right)\right)S$$

(7) $$I{}_{s}\left(I{}_{t}\left(m,n\right)\right)=D{}_{I}\left(I{}_{t}\left(m,n\right)\right)$$

The next step in the morphological operation is to perform erosion and dilation. It is useful in removing small white noises based on kernel size. It also shrinks the mask area. Dilation increases the area of the mask after erosion. Dilation is the process that smooths the edges of the images to be processed further. The erosion and dilation processes are important and contribute to the overall accuracy of detection. The pixels in the isolated area of the image have to be converted to black or white for image processing. The threshold method is used to convert the pixels into black and white. At this point, the mask is obtained and has the values 127 and 255, where 127 is a midtone point and 255 corresponds to white. This range is used to have a smooth value of the masks. The higher the number, the smoother the color transformation obtained. I${}_{Trans}$ is the transformation function for the detection of the threshold value and the formula is shown in Equation (8), where W is White and B is Black pixels, and for the calculation of detection Det(I(m,n)), the formula is shown in Equation (9) where Cont is contour and O${}_{r}$ is the original image and S${}_{i}$ is the segmented image.

(8) $$I_{Trans\left(m,n\right)}=Threshold(I_{s},W_{\left(s,m\right)\left(s,n\right)}\left|\right|B_{\left(s,m)(s,n\right)})$$

(9) $$Det\left(I_{\left(m,n\right)}\right)=Cont\left\{f\left(I_{m}\&I_{t}\right),{(O_{r\left(m,n\right)},S}_{i\left(m,n\right)}\right)\}$$

A bitwise AND operation is implemented between the original image with noise and the mask image, which has been subjected to the binary threshold. Contours are an important indication that show the presence or absence of the cancerous cell. The contours are detected in the image by using the final masks. In the final step, the masks result in contours. Different contours are drawn on the original and the segmented image to ensure that there is a clear distinction between them.

**Figure 1:**
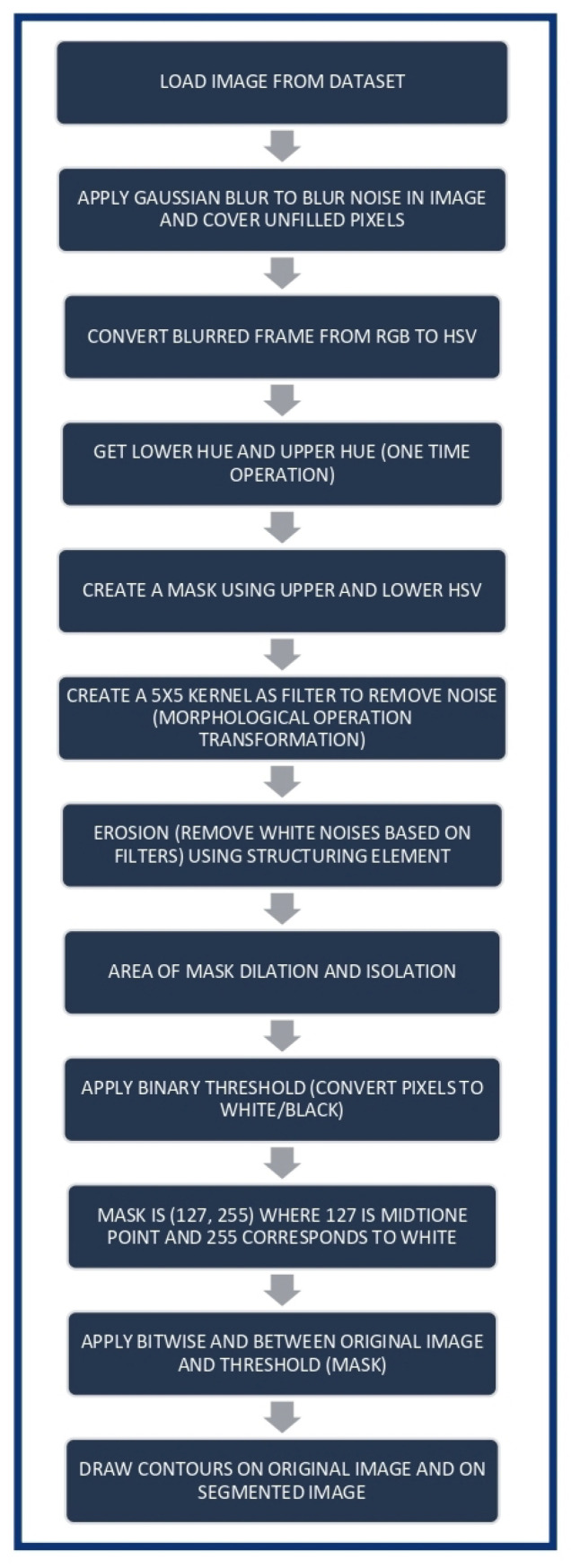
Proposed Method.

**Figure 2:**
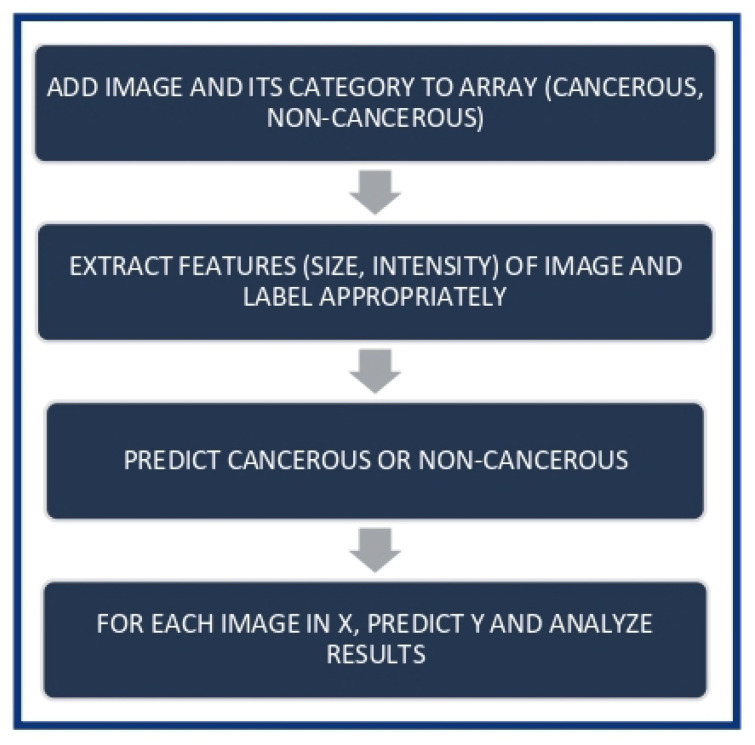
Cancer Cell Detection.

Figure 1 shows the flow diagram of the proposed method and Figure 2 shows the flow diagram for cancer cell detection in which a simple and effective algorithm is used to detect cancerous cells. The image from which the cancer cell has to be detected is added along with its category to the given array. Certain features of the image are extracted and labeled appropriately. The next step is to predict whether the cell is cancerous or not. The prediction values are the final result of the entire algorithm.

### 2.4. Dataset

A combination of two different datasets has been used to evaluate the robustness and accuracy of the proposed method. A total of 81 real-time images (40 ALL images and 41 non-ALL images) are taken and is referred as private dataset in the study for cancer cell detection from Kasturba Medical College (KMC), Mangalore, Karnataka, India, to test the proposed method. The experiments discussed in the literature review section focus on pre-processed images chosen from publicly available datasets. In these datasets, the pre-processing, such as edge smoothing, noise removal, and contrast enhancement, is taken care of. The algorithm designed in the proposed study works on a real-time dataset.

The proposed method is also applied on the ALL-IDB1 dataset, to test whether the adaptiveness of the algorithm of the proposed method can also be applied on publicly available datasets. The proposed method uses 49 cancerous images and 60 non-cancerous images, a total of 109 images from the ALL-IDB1 dataset. The images in the dataset have magnifications ranging from 300–500. The images are labeled ImXXX${}_{Y}$, where Y = 0 if the cells are non-cancerous, or Y = 1 if the cells are cancerous. The images, classified as Acute Lymphoblastic Leukemia affected, can be seen to have blasts with an irregular envelope around the nucleus and have small cavities in the cytoplasm, along with small nucleoli within the nuclei [10].

## 3. Results and Discussion

A confusion matrix is used in this system, as the prediction and classification model can be confused in certain cases—whether to identify the cancer cell or not due to various factors. The prediction values of this confusion matrix are used to check the different ways of finding the efficiency of the developed algorithm.

The False Positive obtained was 3, Total False Negative was 0, True Positive was 40, and True Negative was 38, as shown in Table 2.

The False Positive obtained = 2; Total False negative = 3; True Positive = 46; True Negative = 58, as shown in Table 3.

**Figure 3:**
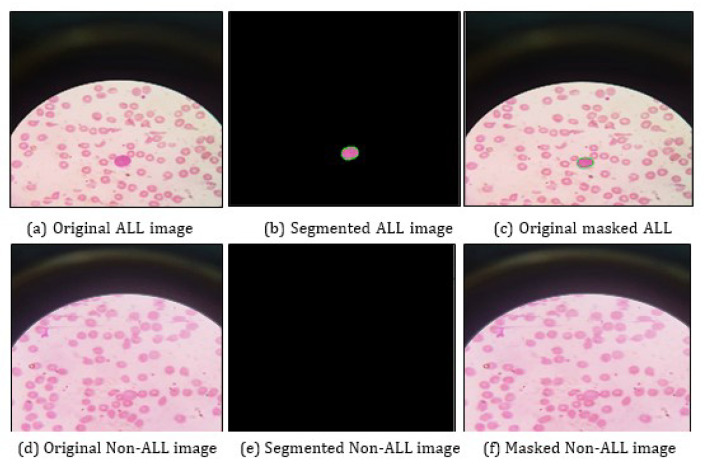
Morphological operations on images from private dataset.

Figure 3 illustrates the morphological operations performed on the Acute Lymphoblastic Leukemia image and Non-Acute Lymphoblastic Leukemia image taken from the real-time dataset. Figure 3a shows the original ALL image with no pre-processing. Figure 3b shows the segmented ALL image, which has contours drawn, highlighting the blast isolated from the rest of the images. Figure 3c shows the original masked ALL image, which has the blast highlighted, with the contours drawn around it. Figure 3d shows the original non-ALL image with no pre-processing. Figure 3e shows a blank for segmentation, as there were no cancerous cells identified by the proposed algorithm. Figure 3f shows the original masked non-ALL image with no segmented part highlighted, as there are no cancerous cells.

**Figure 4:**
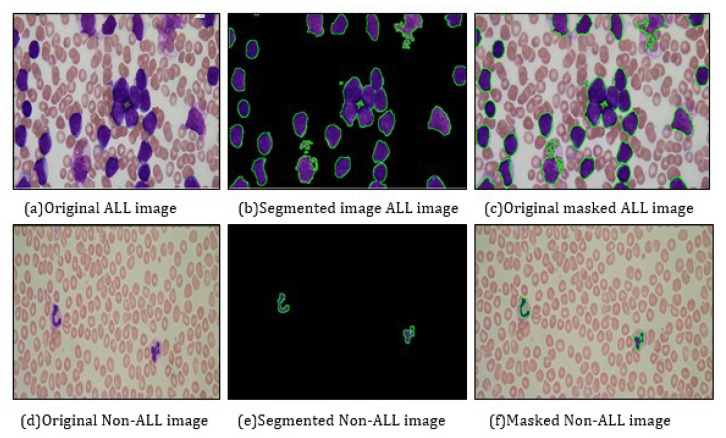
Morphological operations on images from ALL-IDB1 dataset.

Figure 4 illustrates the morphological operations performed on the Acute Lymphoblastic Leukemia image and Non-Acute Lymphoblastic Leukemia image taken from the ALL-IDB1 dataset. Figure 4a shows the original ALL image, with no pre-processing. Figure 4b shows the segmented ALL image, which has contours drawn, highlighting the blast isolated from the rest of the image. Figure 4c shows the original masked ALL image, which has the blast highlighted with the contours drawn around it. Figure 4d shows the original non-ALL image with no pre-processing. Figure 4e shows the segmented non-ALL image with the presence of two contoured shapes highlighted, these shapes correspond to the eosinophils present in the blood sample image. The eosinophils do not represent cancerous blasts. The algorithm identifies them as they are a type of white blood cells. Figure 4f shows the original masked non-ALL image, with the contours highlighted.

The proposed method is evaluated using the standard performance measures.

Table 4 shows the evaluation results obtained when the proposed method is applied on both the private dataset as well as the ALL-IDB1 dataset.

The proposed method is compared with the color thresholding algorithm and Gradient Boosting Decision Tree (GBDT). As shown in Table 5, the proposed method has a higher accuracy of around 4% when compared with the similar color thresholding scheme. Furthermore, when compared with the Gradient Boosting Decision Tree approach, a higher accuracy of around 3% was achieved.

## 4. Conclusions

Cancer is a diseases that is widely prevalent in the world, which does not have a specific cure. Leukemia is a type of blood cancer that prevents the bone marrow from producing healthy blood cells, producing immature white blood cells at an unstoppable and fast rate. Acute Lymphoblastic Leukemia is the highest detected type of leukemia, which can be mostly seen in children. The detection of leukemia is important for providing treatment at an early stage. The proposed GBHSV-Leuk method highlights the importance of the image pre-processing stage for the improved segmentation and classification performance of the algorithm.

The HSV color space is used in the proposed method for the segmentation technique to extract the nucleus, as it yields better results in contrast to RGB color space. The experiment was carried out on a hybrid dataset comprising both a real-time dataset, as well as the publicly available ALL-IDB1 dataset. In the proposed method, the comparison is with two existing methods, Color Thresholding Scheme and Gradient Boost Decision Tree. The comparison results show that the proposed method gives an improved result. The accuracy obtained with the private dataset was 96.30%, while the accuracy obtained with the public dataset was 95.41%. The proposed GBHSV-Leuk technique can efficiently draw contours on multiple cancer cells in the single biopsy image. The application of HSV color model is used in detecting various other types of cancers apart from ALL. 

## Figures and Tables

**Table 1:** Survival rates of leukemia patients.

**Leukemia Type**	**Survival Rate**
Acute Lymphoblastic Leukemia	71.7%
Acute Myelogenous Leukemia	29.4%
Chronic Lymphocytic Leukemia	88.2%
Chronic Myelogenous Leukemia	69.7%

**Table 2:** Confusion matrix for private dataset.

	Predicted Labels	
**Actual labels**	0 (non-cancerous)	1 (cancerous)
0 (non-cancerous)	38	3
1 (cancerous)	0	40

**Table 3:** Confusion matrix for ALL-IDB1 dataset.

	Predicted Labels	
**Actual labels**	0 (non-cancerous)	1 (cancerous)
0 (non-cancerous)	58	2
1 (cancerous)	3	46

**Table 4:** Performance measures evaluated on the two datasets from the observations of the confusion matrix.

	Private Dataset	ALL-IDB1
Accuracy	96.30%	95.41%
Precision score	93.20%	95.83%
Recall Score	100%	87.75%
Specificity	100%	95.81%
F-measure	96.31%	91.61%

**Table 5:** Comparative analysis of proposed method with various methods on ALL-IDB1 dataset.

	Color Thresholding Algorithm	Gradient Boosting Decision Tree	Proposed Method
Accuracy	91.60%	92.50%	95.41%
Precision score	100%	100%	95.83%
Recall Score	83.30%	85%	87.75%
Specificity	100%	91%	95.81%
F-measure	90.88%	91.85%	91.61%

## Data Availability

The ALL-IDB1 dataset is available at the link http://homes.di.unimi.it/scotti/all/#datasets (accessed on 27 May 2020), and access to the private dataset containing 81 images collected from Kasturba Medical College, Mangalore will be given upon request.
